# Differences in the Dominant and Non-Dominant Knee Valgus Angle in Junior Elite and Amateur Soccer Players after Unilateral Landing

**DOI:** 10.3390/sports5010014

**Published:** 2017-02-13

**Authors:** Oliver Ludwig, Steven Simon, Joe Piret, Stephan Becker, Franz Marschall

**Affiliations:** 1 Sportwissenschaftliches Institut, Universität des Saarlandes, Geb B 8.1, 66041 Saarbrücken, Germany; steven.simon1@icloud.com (S.S.); pirleta_1992@hotmail.com (J.P.); f.marschall@mx.uni-saarland.de (F.M.); 2 Fachbereich Sportwissenschaft, Technische Universität Kaiserslautern, 67663 Kaiserslautern, Germany; stephan.becker@sowi.uni-kl.de

**Keywords:** soccer, prevention, dynamic knee valgus, neuromuscular control, single leg drop jump

## Abstract

More than 70% of all knee injuries in soccer occur in non-contact situations. It is known that increased lower limb dynamic knee valgus is associated with such situations. Little has been found out about differences in knee kinematics of the dominant (kicking) and non-dominant (supporting) leg during a single leg landing. A total of 114 male adolescent soccer players (age 14.6 ± 1.1 years) from elite (N = 66) and amateur soccer clubs (N = 48) performed a single leg drop landing down from a box. For each leg, the two-dimensional dynamic knee valgus angle (DKVA) was calculated. Paired *t*-tests were used to statistically determine significant differences between dominant and non-dominant leg DKVA, and *t*-tests were calculated between the two performance groups. Statistically significant differences (*p* < 0.05) were identified for the DKVA between the dominant and non-dominant leg for both amateur and elite players, showing a greater DKVA for the dominant leg. Group differences for the DKVA between amateur and elite players were not found, neither for the dominant, nor for the non-dominant leg. It can be concluded that the non-dominant leg showed more stable dynamics than the dominant leg during unilateral landing regardless of the player’s performance level. This could be due to adaptions to sport-specific requirements. Therefore, it is recommended that programs to prevent knee injuries among soccer players consider the dynamics of each leg individually.

## 1. Introduction

Stabilization disorders in the knee joint in jump landings are associated with injuries of the anterior cruciate ligament (ACL) and patellofemoral pain [1,2]. An enlarged knee valgus in combination with hip adduction and knee flexion seem to increase the probability of a major knee injury [3,4].

In soccer, ACL injuries rank among the most severe injuries involving long periods of convalescence [5,6,7,8,9,10]. Interestingly, most ACL injuries occur in situations without external influence [11,12]. These particularly include quick changes of direction and jump landings [13]. When landing after a jump, such as in header situations, muscular activity must ensure stabilization of the knee joint in all directions [14]. A lack of neuromuscular stability seems to increase the risk of injuries to the knee joint [15,16,17].

Especially in soccer, the dominance of one leg becomes obvious [18]. The neuromuscular requirements of the kicking leg are entirely different from those of the supporting leg [19,20]. It can therefore be assumed that different muscular activity in both extremities may lead to a different degree of muscular stabilization of the knee joint when landing on one leg. Since in men’s soccer the frequency of ACL injuries on the side of the dominant, kicking leg is slightly higher than that on the non-dominant side [21,22], the question arises whether different landing kinematics might play a role in this situation [23]. 

Muscular asymmetries already occur in juniors [24], which might be of medical relevance. Individual balancing exercises are often part of professionals’ training programs. At the same time, the higher training intensity in the professional area can also promote the occurrence of muscular asymmetries resulting in asymmetrical landing kinematics. In previous studies, the dynamic knee valgus angle in the frontal plane has been proven suitable for the analysis of landing kinematics [25,26].

This study is aiming to answer the following questions:

(1) Are there any statistically significant differences between the frontal knee angles of the dominant leg (DOM) and the non-dominant leg (NON) in junior soccer players when they land on one leg?
(2) Are there any differences between the knee angles of the dominant and non-dominant leg in professional and amateur junior soccer players?

## 2. Materials and Methods

A total of 117 male junior soccer players (age: 14.6 ± 1.1 years, height: 171.3 ± 8.2 cm, weight: 59.0 ± 10.6 kg) from seven different junior teams participated in the tests (see Table 1). Three test persons were not included in the evaluation due to wrong or incomplete performances, thus 114 test persons were evaluated. The junior players of the top performance class (N = 66) were members of professional soccer clubs of the German national soccer league (‘Bundesliga’) which run youth academies. They play in the top class of their respective age group and perform at least four soccer-specific training sessions as well as one athletic training each week. In contrast, only two to three training sessions without any specific athletic training are held per week in the amateur clubs (second lowest local league, N = 48).

Exclusion criteria were acute injuries or complaints as well as two-footedness. The participants and their parents or guardians were informed prior to the trial, in accordance with the requirements of the Helsinki Declaration, about the trial objective and trial procedure and gave their written informed consent. The local ethics commission had approved the study.

### 2.1. Test Procedure

The tests in the amateur clubs were carried out on separate dates in the respective club houses. The elite junior players were tested during an international tournament in a mobile test station before the actual tournament matches in a non-exhausted condition. The players of all teams were identically prepared and instructed for the tests. Weight and height were measured on site, and information on leg dominance was provided by both player and coach. The dominant leg was defined as the leg that the player usually kicks with.

**Figure 1:**
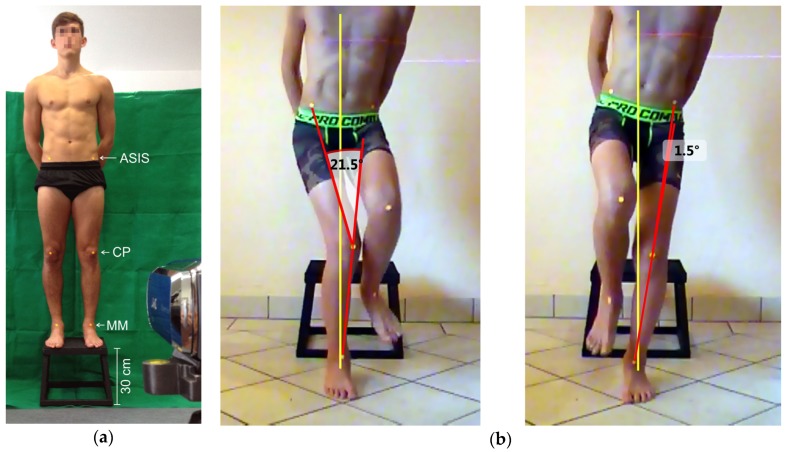
(**a**) Experimental setup: ASIS = marker on the anterior superior iliac spine, CP = marker on the center of the patella, MM = marker on the instep; (**b**) Example of the landing positions of a right-footed soccer player: the dominant (right) leg shows an increased dynamic knee valgus angle (note that the left hip rotates backwards).

The test persons wore sports shorts, no shirt, and were barefoot in order to prevent influences of the shoes on leg kinematics. Marker points with a diameter of 12 mm were fixed to six anatomic landmarks (anterior superior iliac spine (ASIS), middle of the patella (CP), middle of the connecting line between the malleoli on the foot’s instep (MM); Figure 1a). The same person performed palpation and fixed the markers.

Before testing started, a photograph was taken of the test persons in order to measure their natural leg axis, standing with their feet at hip width apart and kneecaps pointing forward.

The players were introduced to the test procedure according to a specific, standardized pattern. The single leg drop jumps were performed from a 30 cm high box (PlyoBox^®^). The arms were crossed behind the back because preliminary measurements showed this to be an appropriate method to eliminate arm swing during the landing process. Otherwise, arm movement would have been an additional uncontrollable parameter as regards trunk stabilization. The drop jump was carried out in a forward direction with both feet, and the landing took place on one foot. A 40 cm × 60 cm mat was placed directly in front of the box to mark the landing zone.

After the standardized instruction, the test person had two trial jumps, the performance of which was corrected, if required. Subsequently, they jumped twice, once landing on the dominant and once on the non-dominant leg. An attempt was deemed a failure and the test had to be repeated if the test person performed an evasive movement when landing (such as a change of the foot position), carried out a balancing movement of the arms, or correction of the jump after landing.

### 2.2. Data Analysis

The image recordings were transferred to a PC and analyzed using the Dartfish 7 Pro Suite (Dartfish, Fribourg, Switzerland) software. For each leg, the time of the highest degree of knee flexion while landing was identified. In the still images, the knee angles in the frontal plane (angle between ASIS, CP and MM; Figure 1a) were calculated for each side. Valgus angles were marked with a positive sign and varus angles were marked with a negative sign. The evaluation distinguished between the dominant (kicking leg) and non-dominant leg (supporting leg).

Data processing and graphical representation were executed using Microsoft Excel 14.1.0. Statistics were calculated by means of XLSTAT 2016 (Addinsoft, New York, NY, USA). To compare the dominant and non-dominant leg, dependent samples *t*-tests were calculated. *t*-Tests were calculated to identify possible differences between the amateur and elite group. Equality of variance was tested in advance, applying Fisher’s F-test, and the degree of freedom was corrected according to Welch-Satterthwaite, if required.

The significance level was set to 5%. The calculated p-values were adjusted according to Bonferroni-Holm. The effect size was determined by Cohen’s d. Values between 0.2 and 0.4 signify weak effects, up to 0.7 they point to medium-sized effects, and if they are >0.7 they imply strong effects.

## 3. Results

There is a statistically significant difference in the dynamic valgus angles when landing on the dominant, kicking leg and on the non-dominant, supporting leg in both the amateur players group (*t*(47) = 2.051, *p* = 0.046) and the elite players group (*t*(65) = 4.001, *p* = 0.000). In the first case, a weak-sized effect occurs, and in the second case, a medium-sized effect occurs (Table 2). The share of left-footed players in the professionals’ group was 29% (19 left-footed vs. 47 right-footed players) and 17% (8 left-footed, 40 right-footed players) in the amateurs’ group. The comparison of the knee angles between the amateur and elite groups does not exhibit any statistically significant differences, neither for the dominant leg (*t*(82) = 1.009, *p* = 0.316) nor for the non-dominant leg (*t*(112) = −0.450, *p* = 0.653).

## 4. Discussion

This study aimed at finding possible differences in the landing kinematics of the dominant and non-dominant leg in junior soccer players. A statistically significant effect of small and medium size was identified in the groups analyzed. The dominant leg showed a larger knee valgus angle than the non-dominant leg, which, at the time of maximum flexion, is generally stabilized in a slight varus position. Figure 1b shows an example of a right-footed soccer player. All subgroups showed large coefficients of variance (Table 2). This was caused by the large inter-individual variation of the knee angles, which might probably have been caused by the individual differences in the players’ static leg axes. The static leg axes and their deviations, such as knock knees (genu valgum) or bowed legs (genu varum) influence the initial position from which a dynamic movement will start. Therefore, further studies should examine the influence of the individual static leg axes on the dynamic valgus angles.

We let the players perform only one jump for each leg after having finished the trial jumps. Although other studies analyzed the mean value of several jumps [25,26], we decided not do so as we wanted to minimize possible learning effects.

Obviously, the kinematic differences reflect the different motor requirements of the supporting and the kicking leg in soccer. Differences between the two extremities can be considered specializations of the sensorimotor system [27]. The supporting leg is optimized in a way to ensure a stable mechanical anchor point for the movement of the kicking leg. This happens through neuromuscular movement programs that dynamically stabilize the foot, knee, and hip joints. The kicking leg’s motion is very complex and depends on the situational motor requirements when guiding or kicking the ball. Simply put, the non-dominant (supporting) leg is optimized for stabilizing the leg axis and hip, also during a one-legged landing. This happens through intensified activation of the hip’s outer rotators and hip abductors [28]. In contrast, the dominant (kicking) leg exhibited a potential instability during our tests when serving as a landing leg. Muscular imbalances between the two sides are known in soccer and are the subject of controversial debate in terms of their manifestation and potential influence on injuries [29,30,31].

Since dynamic knee valgus positions in combination with hip adduction and hip flexion (“medial knee collapse”) seem to promote ACL injuries [23], these kinematic cases must be given special attention. Nevertheless, this study cannot answer the question of whether the increased probability of injury of the knee joint of the dominant leg [22] is also caused by more unfavorable landing kinematics. However, the results show that more research is required in this field in the future.

The differences between the supporting and kicking leg were identified in both groups of players with the effect being larger in the elite player group. On the one hand, it would be plausible if those differences occur more prominently in junior professionals because in their age group, the degree of specialization and training is already very intense, which leads to muscular adaptations [18]. Differences in posture stability when standing on one leg are already known for professional and amateur players [32]. On the other hand, all professional soccer clubs that participated in this study offer a special athletic training at least once a week to compensate for muscular imbalances. Both aspects possibly keep a balance. In any case, the problem of the differences between the two sides concerns both performance groups.

Determining the two-dimensional (2D) knee angle in the frontal plane to assess knee kinematics is well-known from other studies [25,26]. Even though it is impossible to infer the three-dimensional knee angle from the 2D analysis [33], the 2D knee angle still supplies important and usable information [34,35]. Geometrically, this angle is a result of the projection of the knee position into the frontal plane. It therefore includes rotation movement in the hip joint as well as knee flexion, rotation, and adduction [36]. The combination of these movements is known as “dynamic knee valgus” and represents a sum parameter that, in itself, does not say anything about the biomechanical knee valgus [37], but seems to be associated to injuries of the joint. Therefore, this parameter seems to be a time- and cost-efficient measure and supplies useful data on a complex knee movement [35].

Differences between the two extremities during movement exist in many types of sports and are the result of neuromuscular adaptation [38,39]. It is therefore not necessary to consider them basically negative. However, since correlations to injuries are known [40], kinematic and muscular asymmetries should continue to receive their due attention in both amateur and professional training and performance diagnostics. To ensure effective injury prevention, coaches should emphasize not only a suitable warm-up training [15,40,41], but also a separate training of the musculature that stabilizes the knees and hip, which includes coordinate aspects [42,43,44,45,46].

## 5. Conclusions

The differences between the kinematics of the dominant and non-dominant leg during a single leg landing need to be critically assessed, especially in view of injury prevention, and should be reflected in corresponding training programs.

## Figures and Tables

**Table 1:** Anthropometric data of the 144 test persons.

Level	N	Age (years)	Height (cm)	Weight (kg)
**Amateur**	48	15.4 ± 1.4	174.1 ± 6.8	64.1 ± 10.9
**Elite**	66	14.0 ± 0.2	169.3 ± 8.6	55.3 ± 8.8

**Table 2:** Differences in the dynamic knee valgus angles between the dominant (DOM) and non-dominant (NON) leg in the groups examined, CV = coefficient of variation, d = Cohen’s d. Negative values mark varus angles and positive values signify valgus angles.

Level	Leg	df	M	SD	CV	*p*	*d*
**Amateur**	DOM	47	1.419	13.597	9.58	0.046	0.28
	NON	47	−1.856	9.729	−5.24		
**Elite**	DOM	65	3.758	10.036	2.67	0.000	0.64
	NON	65	−2.715	10.281	3.79		
